# Characteristics of the cerebrospinal fluid pressure waveform and craniospinal compliance in idiopathic intracranial hypertension subjects

**DOI:** 10.1186/s12987-018-0106-5

**Published:** 2018-08-01

**Authors:** Monica D. Okon, Cynthia J. Roberts, Ashraf M. Mahmoud, Andrew N. Springer, Robert H. Small, John M. McGregor, Steven E. Katz

**Affiliations:** 1 Department of Biomedical Engineering, The Ohio State University, 1080 Carmack Rd, Columbus, OH 43210 USA; 2 Department of Ophthalmology & Visual Science, The Ohio State University, 915 Olentangy River Rd, Columbus, OH 43212 USA; 3 Department of Anesthesiology, The Ohio State University, 410W. 10th Avenue, Columbus, OH 43210 USA; 4 Department of Neurosurgery, The Ohio State University, 1581 Dodd Drive, Columbus, OH 43210 USA; 5 Ohio Neuro-Ophthalmology, Orbital Disease & Oculoplastics, 3545 Olentangy River Rd, Suite 200, Columbus, OH 43214 USA

**Keywords:** Idiopathic intracranial hypertension, Cerebrospinal fluid pressure waveform, Cerebrospinal fluid pressure pulse amplitude, Craniospinal compliance, Pressure–volume curves, Compliance, Cerebrospinal fluid pressure

## Abstract

**Background:**

Idiopathic intracranial hypertension (IIH) is a condition of abnormally high intracranial pressure with an unknown etiology. The objective of this study is to characterize craniospinal compliance and measure the cerebrospinal fluid (CSF) pressure waveform as CSF is passively drained during a diagnostic and therapeutic lumbar puncture (LP) in IIH.

**Methods:**

Eighteen subjects who met the Modified Dandy Criteria, including papilledema and visual field loss, received an ultrasound guided LP where CSF pressure (CSFP) was recorded at each increment of CSF removal. Joinpoint regression models were used to calculate compliance from CSF pressure and the corresponding volume removed at each increment for each subject. Twelve subjects had their CSFP waveform recorded with an electronic transducer. Body mass index, mean CSFP, and cerebral perfusion pressure (CPP) were also calculated. T-tests were used to compare measurements, and correlations were performed between parameters.

**Results:**

Cerebrospinal fluid pressure, CSFP pulse amplitude (CPA), and CPP were found to be significantly different (p < 0.05) before and after the LP. CSFP and CPA decreased after the LP, while CPP increased. The craniospinal compliance significantly increased (p < 0.05) post-LP. CPA and CSFP were significantly positively correlated.

**Conclusions:**

Both low craniospinal compliance (at high CSFP) and high craniospinal compliance (at low CSFP) regions were determined. The CSFP waveform morphology in IIH was characterized and CPA was found to be positively correlated to the magnitude of CSFP. Future studies will investigate how craniospinal compliance may correlate to symptoms and/or response to therapy in IIH subjects.

**Electronic supplementary material:**

The online version of this article (10.1186/s12987-018-0106-5) contains supplementary material, which is available to authorized users.

## Background

Idiopathic intracranial hypertension (IIH) is a condition of abnormally high intracranial pressure (ICP) with unknown etiology. However, factors such as obesity and stenosis of the venous sinus have been potentially linked [1, 2]. Symptoms include persistent headache, pulsatile tinnitus, diplopia, and visual disturbances such as photophobia [3, 4]. The persistent elevated pressure eventually leads to optic atrophy and vision loss [5]. The management of IIH focuses on the reduction of ICP and ultimately, the protection of vision.

Weight loss, medications, optic nerve sheath fenestrations, and neurosurgical shunting procedures are all therapeutic considerations for the control of intractable headache and the protection of visual function. None of these medical and surgical treatments are curative and they have different risk–benefit profiles. Furthermore, the response to treatment varies between individuals, and there is a lack of consensus in the literature on which intervention is the most effective [6–8]. Aspects of the craniospinal system such as compliance may vary among individuals and thus influence the expression of the disease and response to treatment.

The purpose of this study is to develop a clinical technique to assess craniospinal compliance during the diagnostic lumbar puncture (LP) in IIH. Analysis of the CSFP waveform and the pressure–volume response in IIH will provide information that may assist in management of the disease.

## Methods

Eighteen subjects who presented with signs and symptoms of IIH based on the Modified Dandy Criteria [9] were prospectively recruited under a protocol approved by The Ohio State Institutional Review Board: IRB 2012H0254: long term follow-up of subjects with IIH. Each subject received a standard ophthalmic evaluation by a neuro-ophthalmologist, including visual acuity with a Snellen chart, a slit lamp exam, fundoscopy, and Humphrey Visual Fields (Zeiss Humphrey System, Dublin, California).

Before the LP, all subjects underwent MRI and MRV to rule out structural issues such as mass lesion, infiltrative/inflammatory disease, and venous sinus thrombosis. Each subject subsequently underwent an LP with ultrasound guidance using Siemens Antares Stellar Plus with a CH4-1 transducer (Siemens Medical Solutions, Malvern, PA). An anesthesiologist conducted all of the LPs using either a 4-in. 24-gauge Pencan pencil-point needle, a 4.75-in. 24-gauge Sprotte, or 6-in. 22-gauge Sprotte pencil-point needle to confirm the diagnosis. During the LP, the CSF was passively drained to therapeutically reduce CSFP, in 2–4 ml increments, with a target closing pressure (CP) of 12 mmHg. In 12 subjects, the CSFP waveform was also recorded using an electronic transducer (Edwards LifeScience, Irvine, CA) after each increment of CSF removal.

Measured mean CSFP was plotted against volume removed at each increment for all subjects, with the change in pressure divided by the change in volume representing elastance, which is the inverse of compliance. Löfgren et al. described pressure–volume curves with two compliance regions, a low compliance region at higher CSFP (Region 1) and a high compliance region at lower CSFP (Region 2) [10]. Joinpoint (Joinpoint Regression Program, version 4.5.0.1) is open access software that identifies multiple linear regions in a general dataset, as well as the intersection point they share [11]. This software package was used to determine the two compliance regions in each subject’s dataset for the current study. Based on the Bayesian Information Criterion, Joinpoint calculated a transition point from Region 1 to Region 2 in the pressure–volume curves. This transition point between linear regions was defined as the joinpoint. An example of this method is shown in Additional file 1. The craniospinal compliance in each of these regions was then calculated for all subjects as the absolute inverse of the slope of the pressure–volume regression line in each region. The CSF pressure at the joinpoint from the linear regression model was also recorded.

### Calculations

From the data collected, body mass index (BMI), the mean CSFP, CSFP pulse amplitude (CPA), cerebral perfusion pressure (CPP), and the craniospinal compliance were calculated. BMI was calculated by using the standard method [12]. The mean CSFP was determined as the average between the peak and trough of the CSFP waveform. The CSFP pulse amplitude (CPA) was the difference in pressure at the peak and trough of the CSFP waveform. The cerebral perfusion pressure (CPP) for each subject was the difference between the measured CSFP and the calculated mean arterial blood pressure. The mean arterial blood pressure was calculated as $\left( {pulse\,pressure/3} \right) + diastolic\,pressure$.

Bivariate normal density ellipses for a probability of 0.95 and linear regression analyses were performed between Opening Pressure (OP) and compliance in both regions, between OP and the CSF pressure at the joinpoint, between compliance in Region 1 and compliance in Region 2, as well as between CPA and mean CSFP for each individual subject as well as for the overall population.

T-tests were performed comparing CSFP, CPA, and CPP before and after the LP, as well as between compliance in Region 1 and Region 2, with p < 0.05 as the significance threshold.

## Results

**Fig. 1:**
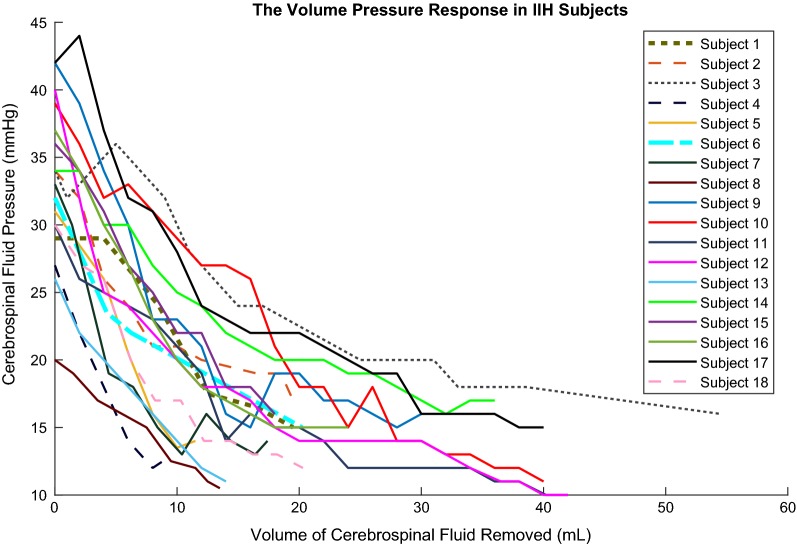
Pressure-Volume curves for all subjects. The measured mean cerebrospinal fluid (CSF) pressure was plotted against each increment of CSF volume removed for all subjects

All subjects were previously undiagnosed, untreated, and were undergoing an LP for diagnosis and possible therapeutic intervention. Each subject’s height, BMI, and the results from the standard ophthalmic evaluation can be found in the Additional file 2. One subject had a BMI less than 25, and thus was not in the overweight or obese category [12]. The Frisén score in subject 1 was not recorded in the chart. Figure 1 shows the relationship between CSFP and CSF volume removal for all subjects. Table 1 summarizes the statistical analysis of the initial and final measurements of CSF pressure, cerebral perfusion pressure (CPP), and CPA, as well as the compliance in Region 1 and Region 2 in all subjects. CSFP, CPA, and CPP were significantly different pre and post LP. CSFP and CPA were all reduced while CPP increased post LP, as expected. The CSFP waveform was not initially studied in Subjects 1–4 because the equipment was not available. Waveforms from subjects 6 and 10 were not recorded due to technical difficulties. Compliance in Region 1 and Region 2 were also found to be significantly different.

**Table 1:** Statistical Summary of the Pre, During, and Post LP

Measurement (during LP)	N	(R1)	(R2)	p value
Compliance^a^ (mL/mmHg)	n = 15	0.70 ± 0.34 (range 0.28–1.5)	3.39 ± 1.84 (range 1.05–7.69)	< 0.0001

Measurement (pre/post LP)	N	Pre-LP	Post-LP	p value
CSFP (mmHg)	n = 18	33.11 ± 5.78 (range 20–42)	13.73 ± 2.44 (range 10–17)	< 0.0001
CPA (mmHg)	n = 12	8.08 ± 2.48 (range 4.37–13.66)	1.29 ± 0.64 (range 0.54–2.29)	< 0.0001
CPP (mmHg)	n = 18	53.22 ± 14.45 (range 30–87)	72.33 ± 10.58 (range 59–103)	< 0.0001

^a^Only subjects with two compliance regions identified by Joinpoint were used in the mean calculation

No joinpoint was identified by the software in three subjects, who were subsequently removed from compliance comparisons and any analysis requiring a joinpoint. In one of these subjects, the opening pressure was 20 mmHg, which is close to the average CSF pressure at the joinpoint of 19.40 ± 3.08 (range 13.26–23.99) mmHg. Therefore, this subject exhibited only Region 2. The other two subjects had insufficient points in either Region 1 or Region 2 for the Joinpoint program to work. The regression lines in Additional file 1 represent elastance, and the mean absolute value of the reciprocal of each, represents compliance. The mean compliance in the first region for the 15 subjects with a joinpoint was significantly lower than the mean compliance in the second region (Table 1).

**Fig. 2:**
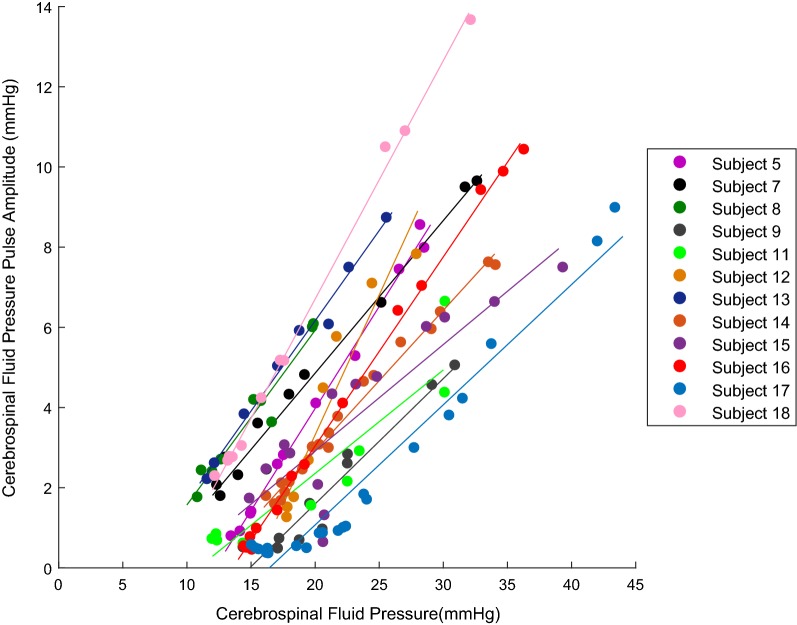
Cerebrospinal fluid pressure pulse amplitude (CPA) with respect to the mean cerebrospinal fluid pressure (CSFP). The CPA and CSFP were positively correlated (p < 0.005) for each individual linear regression analysis of the 12 subjects with recorded waveforms. Note that the strong linear relationship was maintained through both the low compliance region and the high compliance region

The CSFP pulse amplitude showed an overall decrease with passive drainage of CSF (Table 1, Additional file 3). A sample set of recorded waveforms for a single subject is given in Additional file 3 and shows the characteristic reduction of CPA with lowering of CSFP. The CPA and CSFP for the 12 subjects with recorded waveforms were positively correlated (p < 0.005) for each individual linear regression analysis (Fig. 2). The mean of the slopes for the 12 subjects in Fig. 2 was 0.42 ± 0.14 (range 0.26–0.70). The mean of the R^2^ values was 0.94 ± 0.07 (range 0.76–0.998) whereas the overall linear regression analysis for the subjects as a whole population had an R^2^ value of 0.55 with p < 0.05.

The regression between OP and compliance in Regions 1 and 2 was not significant. The regression between OP and the CSFP at the joinpoint was also found to be not significant. BMI and OP were found to be positively correlated (R^2^ value of 0.38 with p < 0.05). The correlation between compliance in Region 1 and Region 2 was also not significant. However, the mean values in each region were found to be significantly different (Table 1).

## Discussion

Studies have used the pressure response to induced cerebrospinal fluid (CSF) volume changes, including the associated pressure waveform, to describe craniospinal elastance and cerebral hemodynamics in multiple forms of hydrocephalus (communicating, non-communicating, and normal-tension), traumatic brain injuries, IIH and healthy subjects [1, 2, 13–17]. Most of these studies assessed the response to increase in ICP through direct bolus injection. In addition, only a few studies have examined craniospinal compliance and cerebral hemodynamics in IIH [1, 18, 19]. These reported experimental techniques do not translate well to a clinically implementable procedure that might be used to assist in the management of IIH. An LP is required to confirm diagnosis in IIH, and injection of fluid would be inappropriate in the presence of increased ICP. As a consequence, the method proposed in the current study can be used to measure the change of CSF pressure (CSFP) with the passive drainage of CSF during a diagnostic LP, which is also used therapeutically to generate a temporary reduction in ICP. The technique of fluid removal has been reported in the literature to experimentally assess compliance in normal pressure hydrocephalus subjects [20]. However, the opening pressure in these subjects was in the normal range, rather than the abnormal range for IIH.

The clinical lumbar puncture used to obtain the diagnostic opening pressure was also used to characterize craniospinal compliance and investigate the CSFP waveform in the current study. The method of passive drainage to calculate compliance was based on all subjects having an initial elevated CSFP. In Region 1, a small change in CSF volume generated a large change in measured CSFP. In Region 2, a large change in CSF volume generated a small change in measured CSFP. The target pressure of 12 mmHg was not reached in some subjects, as the Region 2 pressure stabilized at a higher level, even as CSF continued to be drained. Therefore, the LP was concluded once Region 2 was well established and CSFP did not change further. All subjects had CSFP reduced below 16 mmHg.

Analyzing the pressure volume curve with two linear regions, as discussed by Löfgren in dogs, was chosen for the current study due to the nature of the pressure–volume relationship associated with our IIH subjects [10]. Löfgren’s study used a pressure range that was broader than other studies and characterized the composite pressure–volume response as a function of both cranial and spinal responses [10, 21]. In addition, Anile and Kasprowicz have shown that craniospinal response is viscoelastic [13, 22]. The shape of the pressure–volume curve would be affected by whether fluid is added or removed. Smielewski discussed how bolus manipulation, constant infusion, lumbar ventricular perfusion, and constant pressure infusion may induce a vasomotor response which can disturb a pressure reading [20]. To our knowledge, there are no current human models that measured a pressure–volume curve generated by passive drainage of CSF from an abnormally high CSFP, as in IIH. Previous studies have shown an exponential rise in CSFP with a bolus injection of fluid [17, 23]. A limitation of the current study is the lack of knowledge regarding the repeatability of this technique to calculate compliance. However, treatment is designed to lower intracranial pressure and therefore it may affect compliance, so the pressure–volume curve would be expected to be different.

Other investigators have focused their efforts on measuring compliance noninvasively through models based on MRI measurements and anatomical changes [1, 24–26]. However, these efforts to make the measurement non-invasive would be more helpful after a baseline has been established. One study found reduced compliance in IIH when compared to healthy subjects using MRI [32]. However, the reduced compliance in IIH can be expected because CSFP is presumably higher than in normal subjects. The current study utilizes the diagnostic lumbar puncture as an avenue to characterize an individual’s craniospinal compliance. This method quantifies an individual’s cerebrospinal system response, and may help clinicians to better tailor IIH disease management. The measurement of craniospinal compliance in IIH may provide clinical benefit by evaluating the cerebrospinal system’s ability to adapt to changes [27]. The ability to respond to changes in the cerebrospinal system may lead to differences in the manifestation of symptoms or responses to treatment.

Previous studies have shown that the amplitude of pulsations in the CSFP waveform can be influenced by compliance, the magnitude of CSFP, and cerebral blood flow [15, 28–31]. Szewczykowski, Avezaat, Czosnyka, and Qvarlander found a positive relationship between the overall CPA and mean value of CSFP waveform in subjects with and without CSF disorders, which is also consistent with the data reported in the current study, and shown in Fig. 2 [32–37]. However, some previous studies have also reported a region of constant compliance below 10 mmHg [32, 35–37]. None of our subjects were evaluated in this region, since the target closing pressure for the lumbar puncture was 12 mmHg. This target was not achieved in several of our subjects where the CSFP leveled above 12 mmHg and did not reduce in this region even with passive fluid removal.

Additional file 3 shows the influence of CSF drainage on CSFP and craniospinal compliance as it inversely affects the overall amplitude of the CSFP waveform pulsations. As cerebral perfusion pressure increases, the pulse amplitude decreases, due to a change in compliance. Eide et al. reported that all of their IIH subjects who were undergoing a shunt placement had an elevated pulse amplitude (above 4 mmHg) despite having a normal ICP level [38]. The CPA in those subjects ranged from 4–8.7 mmHg [38]. Eide measured the ICP waveform in the frontal brain parenchyma while the current study measured in the lumbar region.

It is interesting to note the change in waveform morphology as CSFP is reduced and compliance is increased, as illustrated in Additional file 3. The pulsatile nature is attributed to the arterial and venous pulsations [30], and the CSFP waveform directly reflects cardiovascular events. At the highest CSFP where compliance is low, the dicrotic notch is clearly visible, similar to an arterial waveform [31]. As CSF volume was removed, the CSFP was reduced and the morphology of CSFP waveform also changed. When the CSFP was lowered to a normal range (< 20 mmHg), the distinct dicrotic notch in the waveform disappeared. Thus, the distinct features of the arterial waveform are transmitted to the CSF system when it is in a low compliance state, but not in a high compliance state.

## Conclusions

The objective of the current study was to develop a clinically implementable technique for characterizing the CSFP waveform and craniospinal compliance in IIH. This objective was met using passive drainage of CSF during the diagnostic lumbar puncture, rather than bolus injection. Regions of low and high compliance were reported that corresponded to high CSFP and low CSFP, respectively, as well as a CSF pressure where a transition between the two regions occurred. CSFP magnitude, craniospinal compliance, and cerebral hemodynamics influence the CSFP waveform measured while using a technique that is clinically feasible. These parameters may predict the cerebrospinal system’s ability to adjust to induced changes. The next step would be to investigate whether such parameters can be associated to severity of symptoms and response to treatment in IIH.

## Additional files

**Additional file 1.** A Pressure-Volume curve (Subject Two) illustrating the two elastance regions. Compliance (inverse of elastance) in Region 1 and Region 2 calculated were 0.61 and 3.85 mL/mmHg, respectively. The starred points indicate the high compliance region.

**Additional file 2.** The presenting Humphrey Visual Field, Frisén Score, height and body mass index for all the subjects in the study.

**Additional file 3.** An example is shown of a series of CSF pressure (CSFP) waveforms of a single subject, measured with the passive drainage of CSF during the lumbar puncture procedure. As CSF pressure was reduced in this subject, the CSF pressure pulse amplitude was also reduced.

## Abbreviations

BMI: body mass index
CP: closing pressure
CPA: CSFP pulse amplitude
CPP: cerebral perfusion pressure
CSFP: cerebrospinal fluid pressure
ICP: intracranial pressure
IIH: idiopathic intracranial hypertension
LP: lumbar puncture
OP: opening pressure
