# Supplementary figures and images for: Characteristics of the cerebrospinal fluid pressure waveform and craniospinal compliance in idiopathic intracranial hypertension subjects
Source: Fluids Barriers CNS. 2018 Aug 1;15:21. doi: 10.1186/s12987-018-0106-5 (PMC6069551; doi:10.1186/s12987-018-0106-5)

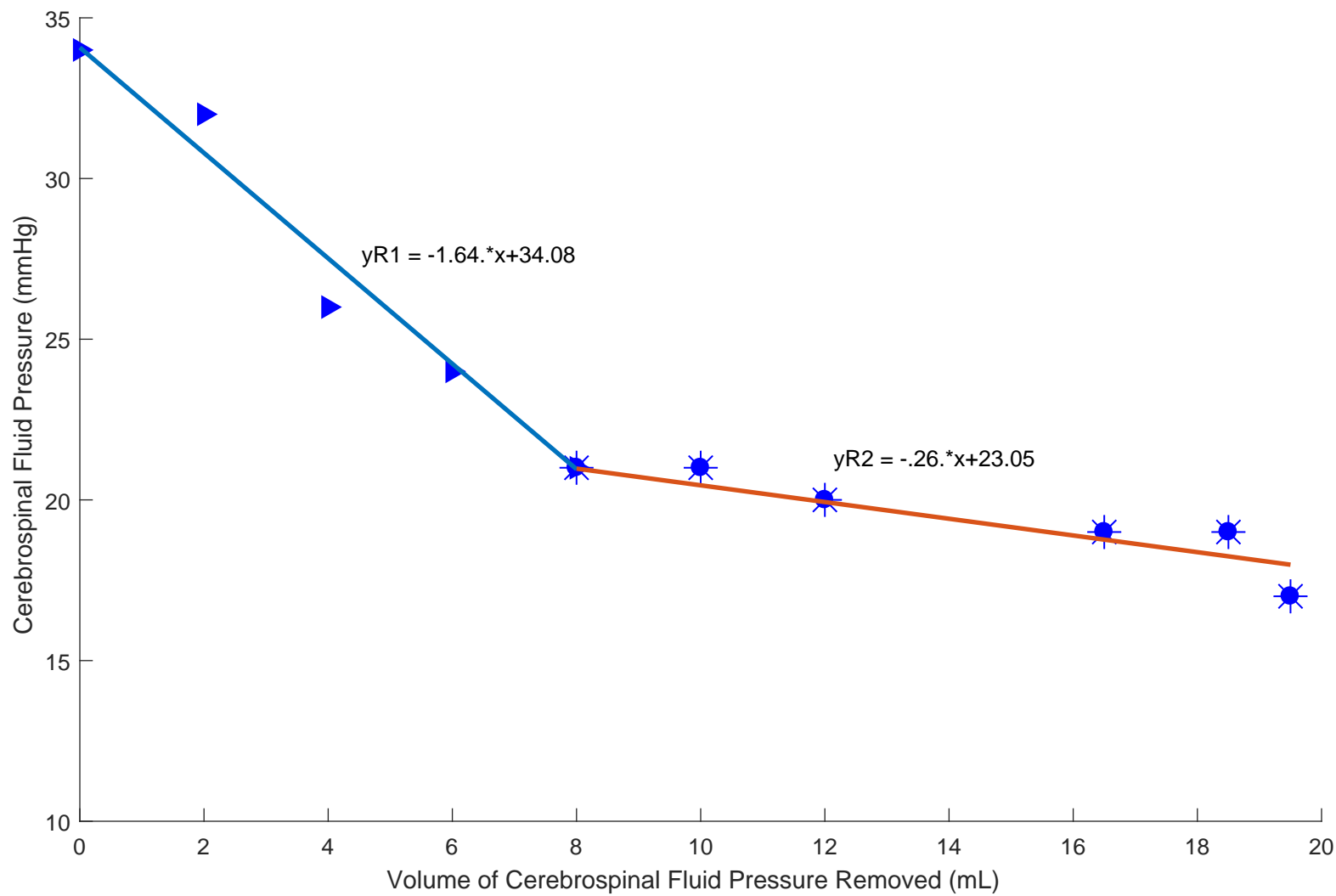

Supplement: Supplementary file 1 — Additional file 1. A Pressure-Volume curve (Subject Two) illustrating the two elastance regions. Compliance (inverse of elastance) in Region 1 and Region 2 calculated were 0.61 and 3.85 mL/mmHg, respectively. The starred points indicate the high compliance region. [file 12987_2018_106_MOESM1_ESM.pdf]

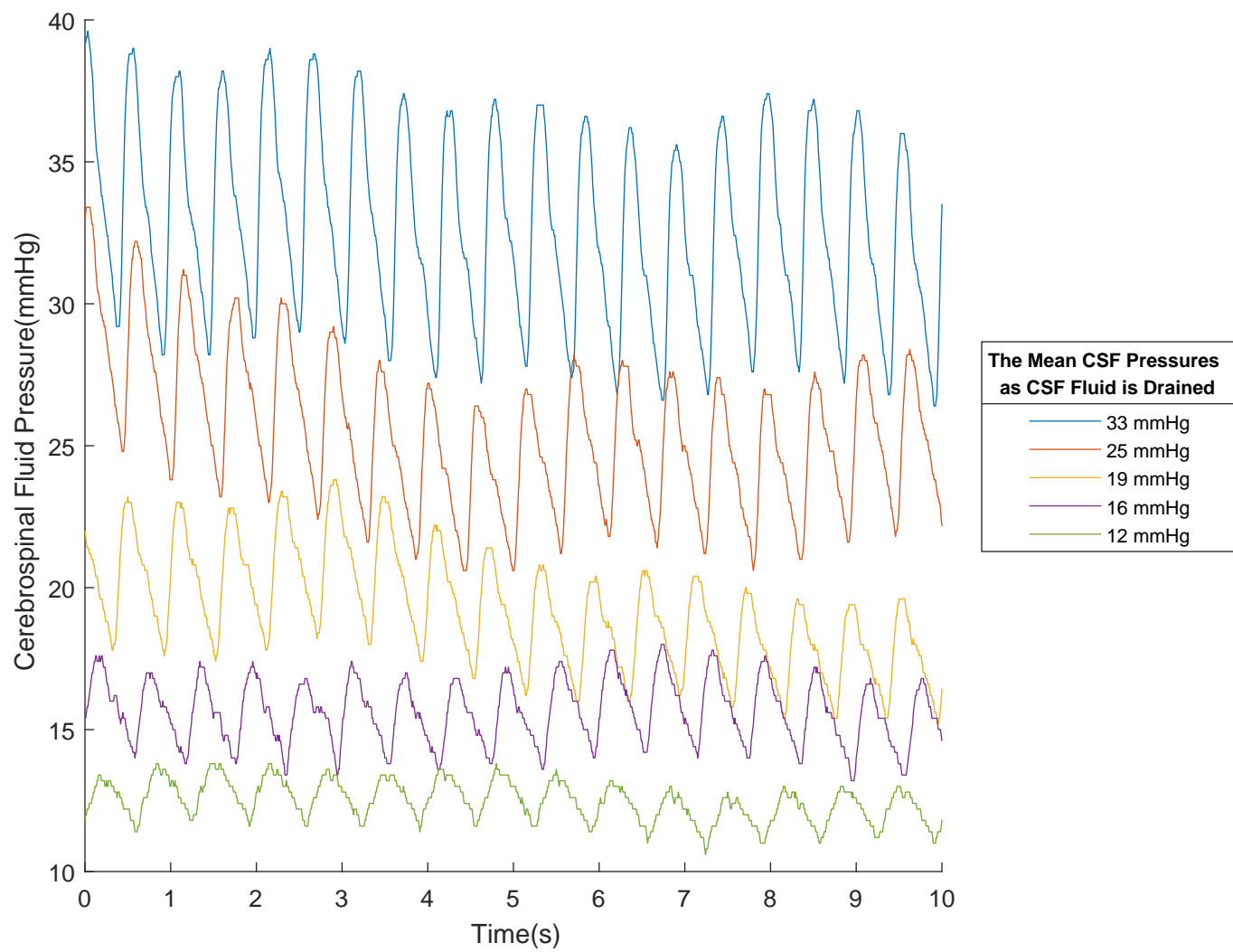

Supplement: Supplementary file 3 — Additional file 3. An example is shown of a series of CSF pressure (CSFP) waveforms of a single subject, measured with the passive drainage of CSF during the lumbar puncture procedure. As CSF pressure was reduced in this subject, the CSF pressure pulse amplitude was also reduced. [file 12987_2018_106_MOESM3_ESM.pdf]
