# Supplementary material for: Characteristics of the cerebrospinal fluid pressure waveform and craniospinal compliance in idiopathic intracranial hypertension subjects
Source: Fluids Barriers CNS. 2018 Aug 1;15:21. doi: 10.1186/s12987-018-0106-5 (PMC6069551; doi:10.1186/s12987-018-0106-5)
Supplement: Supplementary file 2 — Additional file 2. The presenting Humphrey Visual Field, Frisén Score, height and body mass index for all the subjects in the study. [file 12987_2018_106_MOESM2_ESM.docx]

Additional file 2: Ophthalmic Evaluation for Each Subject

| **Subject** | **Eye** | **Presenting Humphrey Visual Field**  **(Mean Deviation)** | **Presenting Frisén Score** | **Height (meters)** | **Body Mass Index**  **(kg/m^2^ )** |
| --- | --- | --- | --- | --- | --- |
| 1 | OD | 19.2 | Not Available | 1.63 | 35 |
|  | OS | 1.69 | Not Available |  |  |
| 2 | OD | 33.02 | 4 | 1.73 | 45 |
|  | OS | 33.96 | 4 |  |  |
| 3 | OD | 6.24 | 1 | 1.7018 | 67 |
|  | OS | 4.25 | 1 |  |  |
| 4 | OD | 3.19 | 1 | 1.6256 | 30 |
|  | OS | 4.95 | 1 |  |  |
| 5 | OD | 6.31 | 4 | 1.7526 | 29 |
|  | OS | 6.72 | 4 |  |  |
| 6 | OD | 2.95 | 1 | 1.6002 | 35 |
|  | OS | 1.85 | Tr |  |  |
| 7 | OD | 6.58 | 4 | 1.6764 | 24 |
|  | OS | 6.02 | 4 |  |  |
| 8 | OD | 0.8 | 0 | 1.6256 | 30 |
|  | OS | 1.77 | 0 |  |  |
| 9 | OD | 6.51 | 4 | 1.6764 | 50 |
|  | OS | 6.35 | 4 |  |  |
| 10 | OD | 2.29 | 2-3 | 1.651 | 50 |
|  | OS | 2.36 | 3 |  |  |
| 11 | OD | 3.18 | 3 – 4 | 1.8034 | 34 |
|  | OS | 3.44 | 4 |  |  |
| 12 | OD | 2.56 | 2-3 | 1.6256 | 36 |
|  | OS | 5.86 | 2-3 |  |  |
| 13 | OD | 1.44 | 1-2 | 1.6256 | 26 |
|  | OS | 1.95 | 1-2 |  |  |
| 14 | OD | 0.99 | 1 | 1.5494 | 50 |
|  | OS | 0.93 | 0-1 |  |  |
| 15 | OD | 4.82 | 3-4 | 1.7526 | 35 |
|  | OS | 3.67 | 3 |  |  |
| 16 | OD | 9.72 | 1 | 1.6256 | 59 |
|  | OS | 13.31 | 1 |  |  |
| 17 | OD | 10.81 | 5 | 1.5748 | 56 |
|  | OS | 9.39 | 5 |  |  |
| 18 | OD | 1.51 | 1 | 1.6002 | 28 |
|  | OS | 0.26 | 1 |  |  |
